# A Case of Fracture Blisters: A Major Bullae in the Road to Definitive Care

**DOI:** 10.7759/cureus.91525

**Published:** 2025-09-03

**Authors:** Samantha R Steiss, Shikshya Baral

**Affiliations:** 1 Dermatology, Edward Via College of Osteopathic Medicine, Blacksburg, USA; 2 Internal Medicine, Sentara Northern Virginia Medical Center, Woodbridge, USA

**Keywords:** barrier repair, delayed surgical intervention, fracture blisters, high-energy trauma, negative pressure wound therapy (npwt-id), orthopedic complications, serosanguineous blisters, serous blisters, trimalleolar ankle fracture, wound management

## Abstract

Fracture blisters are a rare yet serious complication of high-energy trauma injuries. In this report, we present the case of a 55-year-old woman who sustained a trimalleolar ankle fracture and subsequently developed extensive serous and serosanguineous blisters on the overlying skin. These fracture blisters significantly delayed her definitive surgical intervention, highlighting the challenges in managing this complication. This case exemplifies the importance of early recognition and appropriate management of fracture blisters to minimize delays in treatment and potential complications.

## Introduction

Fracture blisters are a rare complication of high-energy trauma, occurring in approximately 2.9% of all acute fractures requiring hospitalization [1,2]. They typically develop six to 72 hours after the inciting injury and overlying an acute elbow or ankle fracture where there is less soft tissue surrounding the bones [1,3]. Additional risk factors include conditions that impair wound healing, such as peripheral vascular disease and diabetes mellitus [2]. Fracture blister formation is attributed to separation at the dermal-epidermal junction, and the blister fluid may be either serous, hemorrhagic, or mixed (serosanguineous) [4]. Blood-filled blisters typically represent a more severe injury, as there is complete separation of the dermal-epidermal junction [5]. In contrast, serous-filled blisters indicate only partial separation of the dermal-epidermal junction [5]. The accumulation of fracture blister fluid is attributed to the opening of the paracellular pathway, resulting from a significant reduction in the expression of tight junction proteins, including claudins and occludin [6]. The reduction of tight junction proteins decreases the skin's barrier function, making it more permeable [6]. Guo et al. suggest that this reduction in tight junction expression may serve as an explanation for fracture blister formation and the decrease in compartment pressure seen beneath blistered skin in severe acute fractures of the tibial plateau.

At this time, there is a lack of comprehensive guidelines for the management of fracture blisters [4]. Although it is minimal, current evidence suggests that incisions through fracture blister beds, especially hemorrhagic type, are associated with an increased risk of infection [4,5]. The majority of blisters demonstrate a sterile aspirate, but a small percentage are colonized with bacteria prior to rupture [5]. However, no specific predicting factors for positive aspirate or evidence that positive aspirate increases risk for infection have been identified [5]. Nonetheless, fracture blisters are known to be associated with increased infection rates and wound breakdown [6]. This case serves to highlight the importance of accounting for fracture blisters as a potential complication of high-energy trauma and emphasizes the need for evidence-based management strategies to reduce delays in definitive treatment.

## Case presentation

A 55-year-old African American woman presented to the emergency department (ED) with generalized left ankle pain following a traumatic fall. She denied any additional symptoms. Her medical history included deep vein thrombosis and insulin-dependent diabetes mellitus. Vital signs were blood pressure, 158/87 mmHg; heart rate, 113 beats per minute; respiratory rate, 18 breaths per minute; temperature, 97 ℉; and SpO2, 94% on room air. Physical examination revealed left ankle tenderness and deformity. 

**Figure 1:**
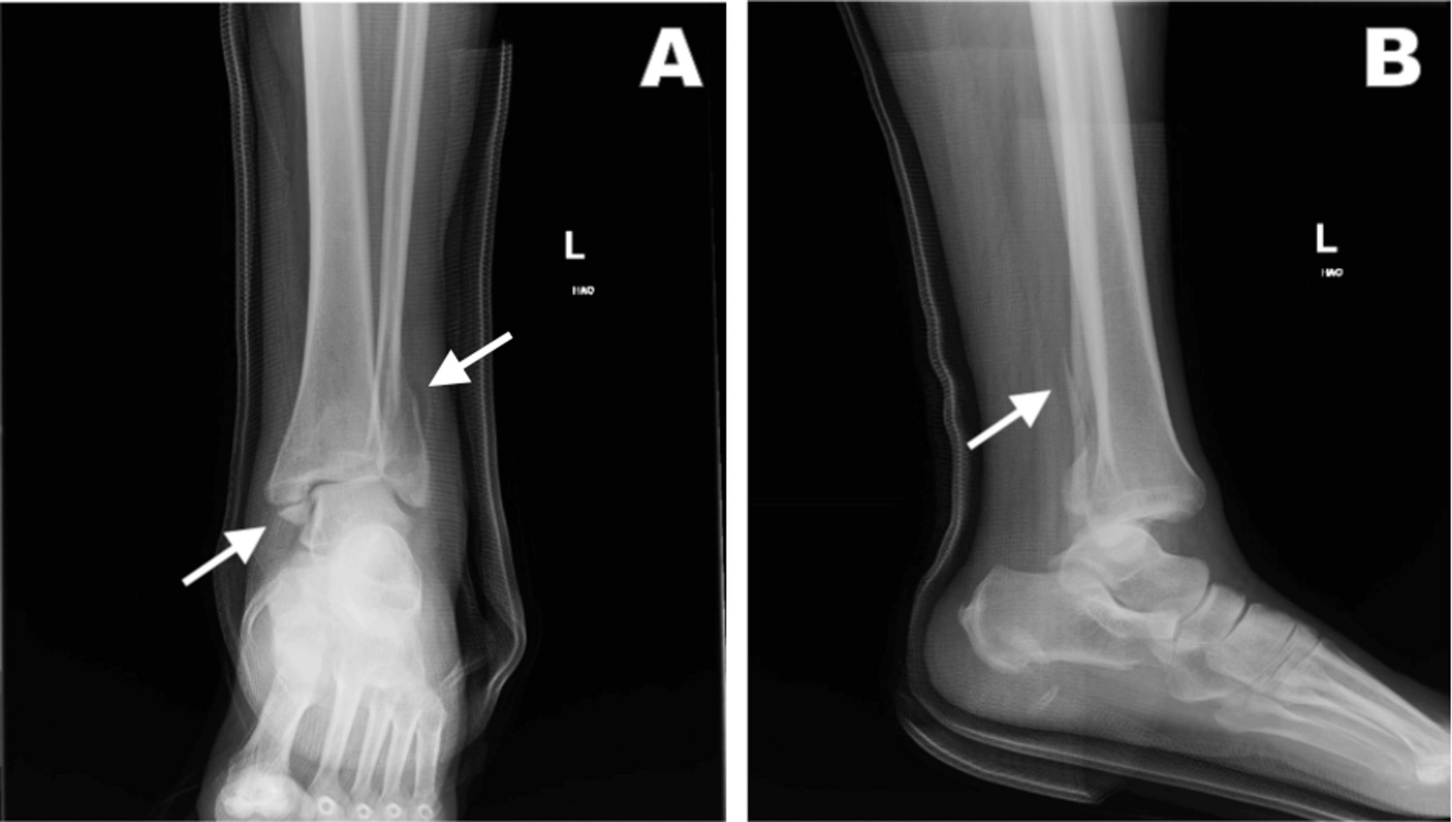
Plain radiographs of the patient's left ankle prior to closed reduction. (A) Anterior-posterior view. (B) Lateral view.

Plain radiographs of the left ankle displayed a closed trimalleolar fracture of the left ankle with lateral and posterior dislocation at the tibiotalar joint (Figure 1). The patient's ED treatment included closed reduction, splinting, and pain management. Upon discharge, instructions for follow-up with orthopedics for definitive treatment were provided. 

**Figure 2:**
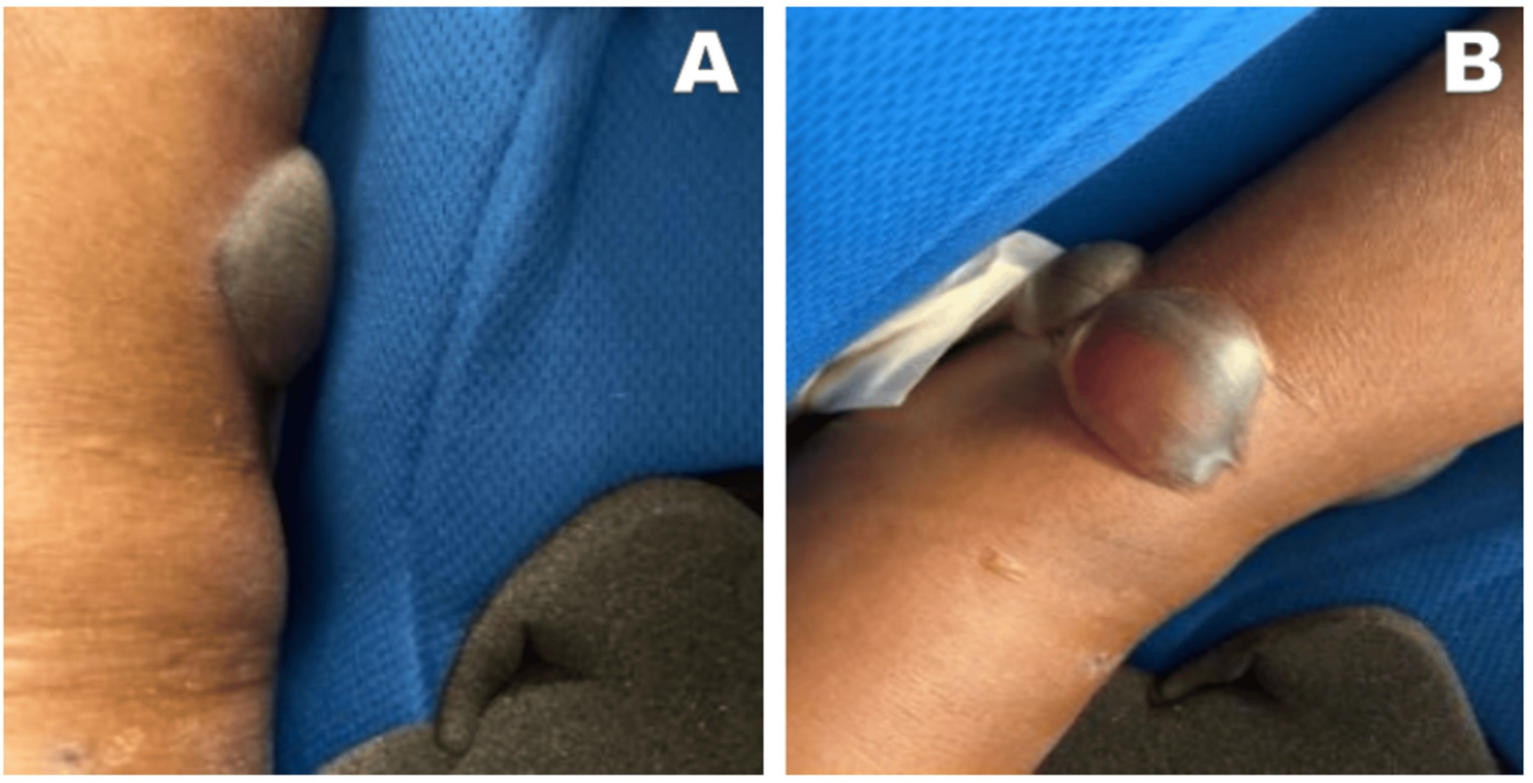
Fracture bullae appearance on the patient's left ankle, nine days status post-traumatic fall. (A) Left lateral ankle. (B) Left medial ankle.

However, two days later, the patient returned to the ED presenting with an inability to ambulate, increased left ankle swelling, and the development of three golf-ball-sized fracture blisters of the serous and serosanguineous type around her left ankle. She was admitted for further management, which included strict bed rest, left lower leg elevation, and left ankle stabilization in an orthopedic boot. The bullae were kept intact, cleansed daily with Vashe (a hypochlorous acid wound solution), and dressed with Hydrofera Blue (an antibacterial foam wound dressing) for protection (Figure 2). Her hospitalization was complicated by new-onset atrial fibrillation and pulmonary embolism. She was rate-controlled and received anticoagulation with Eliquis (apixaban).

**Figure 3:**
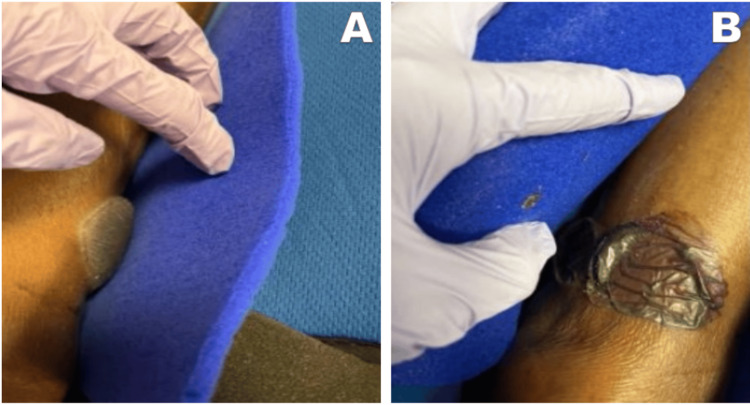
Fracture bullae appearance on the patient's left ankle, 16 days status post-traumatic fall. (A) Left lateral ankle. (B) Left medial ankle.

Given the extent of the bullae, the orthopedic team deferred surgery to avoid any additional complications. Ultimately, surgery was completed once the blisters were mostly resolved, a total of three weeks after their initial presentation (Figure 3).

## Discussion

The most significant implication of fracture blisters is the clinical obstacle they present to the treatment of the underlying fracture, as they may significantly delay the time to definitive fixation compared to patients without fracture blisters [1,5]. Korrapati et al. found that the average time to definitive surgery in patients with fracture blisters was 14.2 days. In contrast, the average time to definitive surgery in patients without fracture blisters was 7.9 days [1]. Due to the complications and poor outcomes associated with fracture blister development, current guidelines favor a staged approach for the orthopedic management of these cases [1]. This approach includes an initial external fixation to stabilize the fracture without extensive insult to the surrounding soft tissue [1]. Then, once the overlying skin has re-epithelialized, the fracture is repaired utilizing a staged open reduction internal fixation -- a two-step surgical approach aimed at minimizing soft-tissue complications and infection rates [1]. 

The extensive delay in this patient's definitive care (21 days) represents the importance of considering the complication of fracture blisters, especially in acute fractures of the ankle, wrist, and elbow [2,7]. Prompt consideration of this rare dermatological complication could influence swift intervention and avoid prolonged postponement of definitive treatment [8]. 

Despite the known obstacles that fracture blisters create, there has yet to be a clear consensus on the optimal management of fracture blisters [1,4]. Currently, physicians utilize a variety of treatment methods to care for fracture blisters, but the most common treatment modalities include normal saline dressings, paraffin gauze, and betadine-based dressings [4]. Despite the various treatment methods utilized, they share a common goal of reducing tissue edema and inflammation [4]. Additional management techniques, including cryotherapy, limb elevation and rest, splinting, limited external fixation, large blister aspiration and de-roofing, and local antimicrobial dressing, are evidence-based practices supported by the current literature on this topic [4]. However, management of fracture blisters with systemic antibiotics and steroids should be avoided, unless otherwise clinically indicated, as their use has not been sufficiently supported [4]. 

In a recent study, circumferential negative pressure wound therapy with sterile saline instillation (NPWT-id) demonstrated promising results as a preoperative management option for patients with blisters overlying closed fractures [9]. Utilization of the NPWT-id technique may decrease the time to reepithelialization, shorten the delay of surgical repair, minimize the need for alternative surgical approaches, and reduce post-operative wound complications [9]. Barrier repair products, such as ceramides and hyaluronic acid, represent an additional category to be considered in the management of patients with fracture blisters, both pre- and post-operatively, as they create an environment optimal for healing by mimicking physiologic lipids and acting as a humectant [10].

## Conclusions

As highlighted in this report, the complication of fracture blisters is a rare yet serious complication of acute high-energy trauma fractures. Overall, this case represents the need for an evidence-based consensus on the acute management of fracture blisters, balancing prevalent dermatological and orthopedic considerations. This consensus would allow physicians to minimize additional complications and time to definitive treatment for patients who develop fracture blisters.
